# Electricity and Its Use in Medicine

**Published:** 1894-04-07

**Authors:** S. W. Wheaton

**Affiliations:** Physician Royal Hospital for Children and Women


### Medical Progress and Hospital Clinics.

[*The Editor will be glad to receive offers of co-operation and contributions from members of the profession. All letters should be addressed to* The Editor, The Lodge, Porchester Square, London, W.]

### ELECTRICITY AND ITS USE IN MEDICINE.

### I.—The Effect upon the Body in Health.

By S. W. Wheaton, M.D. Lond., M.R.C.P., D.P.H., Physician Royal Hospital for Children and Women.

The human body may be considered, from an electrical point of view, as an extremely good conductor, which readily allows of the passage of electricity, but which is enclosed completely by the skin—which is a non-conductor. The interior of the human body is a good conductor, because the tissues are either liquid, as in the case of the blood, or bathed in saline fluid, as in the case of the muscles. It has been proved that a dried mummy will not allow of the passage of electricity through it, even if the needles conveying the current are thrust deeply into it. In the case of the skin its insulating power is chiefly due to the fact that it is dry, and if it be soaked in water, or salt and water, it becomes a much better conductor. When electricity passes through any body it experiences more or less resistance to its passage, and this is greater or less according as the body is a bad or good conductor. This resistance may be compared with the friction which any fluid would experience in passing through a pipe. A good conductor is like a pipe with a large diameter, and a bad conductor, or insulator, is like a pipe of very small diameter. The longer the pipe or conductor the greater the friction or resistance to the current. Friction produces heat (as in the familiar example of the small boy rubbing the button on his coat), and so does electrical friction or resistance. I will drawn attention to this fact later on. Now when we pass a current through the body it will experience resistance in its flow, and the resistance of the body, including the skin which has been soaked in salt and water, is found to be from 1,100 to 1,200 ohms, from the hand across the body to the opposite foot. By this we mean to say that it is equal to the resistance of 1,100 to 1,200 columns of mercury, 1 square millimetre in section and 1·04 metres long, placed in contact, the resistance of one such column of mercury being called an ohm. The late Dr. Stone was the first to investigate this point thoroughly, and to make accurate determinations. I have repeated his experiments, and can confirm his conclusions as to this resistance. Some interesting results have followed these experiments, and were partly ascertained by Dr. Stone himself. Metals are good conductors; therefore if the body is impregnated with metal, such as lead or mercury, as in cases of lead or mercurial poisoning, it would be expected to conduct better than before, and its resistance would be diminished. This deduction is found to be true. The resistance of the human body is also less in dropsy, owing to the soaking of the tissues in saline fluid, which is especially marked in the case of the skin, and causes it to conduct better than in health. It is increased, on the other hand, in hemiplegia, on the paralysed side. From these considerations it was thought that the estimation of the resistance of the body would be valuable as a means of diagnosis; but there are many fallacies to be overcome. Dr. Cardiew found the resistance of the body to be diminished in cases of exophthalmic goitre; but the skin conducts better when moist because its resistance is less, and the lowered resistance in this disease was found to be explained by the constant soaking of the skin in the profuse perspiration which is so constant a feature of the disease. The skin also varies in thickness in different people. The resistance of a man of 6 ft. 2 in. in height was found to be less than that of a man of 5 ft. 4 in., probably because the tall man had a thinner skin. The amount of moisture in the air must also affect the resistance of the skin, and dampness will lessen it by soaking it and making it conduct better; also damp air itself is a good conductor. In a very dry atmosphere, such as that of some parts of North America, electricity accumulates on the surface of the body owing to the insulating action of the dry air, and a few rubs with the feet on the carpet will generate enough to enable anyone to light the gas with a spark from his finger; the spark being produced by the electricity rushing out of the body to recombine with that of the earth through the metal pipe, which is a good conductor. Another familiar example of the same thing is the production of sparks by rubbing a cat's back with the dry hand in dry weather.

Another action of the electric current on the body besides the development of heat and resistance, is that of electrolysis, by which is meant the decomposition of liquids by an electrical current. The saline fluid in which the tissues are bathed is broken up by very strong currents into free acid, which appears at one pole, and oxygen, or an oxide, which appears at the other. This is the foundation of the treatment of aneurism by electrolysis, the blood is caused to clot from the heat which is produced, and also by the free acid which is produced coagulating its albumen. In the electrolysis of nævi, bubbles of gas can be seen to be given off at the negative pole. The fourth action is that of osmosis, by which is meant the transference of substances from one pole to the other through the tissues. In this way, the current has been used to convey lithia from the surface of the skin to gouty concretions, where it would combine with the uric acid composing these concretions to form urate of lithia, and urate of lithia being very soluble would be carried away in the blood. In the same way iodide of potassium can be administered locally. The fifth effect is that of causing dilatation of the vessels of the skin; in this way bringing more blood to the part to which it is applied. In this action on vasodilator nerves it only resembles heat, irritants, and other stimuli. The sixth effect is that of causing contraction of muscle fibre. The main facts relating to this effect are, that a continuous current in healthy muscle causes contraction only when it is started or stopped, and a current which is rapidly interrupted or broken causes the muscle to pass into a tetanic condition. Here we may notice the fact that the current passed along a nerve produces the same effect as a nervous impulse. If the current passes along a motor nerve, the muscle supplied by this nerve will contract; if it passes along a sensory one it will cause a sensation identical with that produced by ordinary stimulation of the nerve. For instance, stimulation of the optic nerve causes a sensation of light, of the auditory, noises; and so on. Lastly, Ferrier has shown that electrical stimulation of certain parts of the brain cortex produces definite localised movements, according to the part stimulated. This brings us to the oft-debated question whether nerve force and electricity are identical, and as to the truth of the inscription, "Electricity is life?" With regard to the first question, I may say that nerve force and electricity are possibly identical. The identity of effects produced by stimulation of a nerve by electricity, and by nervous impulse; the fact that currents are always present in nerve and muscle, that all other stimuli fail when applied to the brain surface to excite those movements before mentioned; and that the torpedo fish is able to produce an electric discharge by means of nerves passing from its brain to certain organs; all point strongly to the identity of nerve force and electricity. Recent observations have shown that both electrical and nervous impulses travel at the same rate in a nerve. With regard to the last query, electricity is certainly not life, but its generation in the form of nervous impulses is perhaps one of the functions of life.

I wish now to draw attention to the effects of extremely powerful currents upon the body. These effects form a new class of accidents with which we as medical men have to deal, and they generally occur in connection with electric lighting, in which light is produced by rendering bodies incandescent, owing to the high resistance which they oppose to the passage of powerful currents. These cases occur in many instances owing to the insulating material covering the conductors being removed, and a man touching the exposed conductor with his hand or other part of his body. Under these circumstances the current takes the shortest course, where it meets with least resistance, and returns through the body of the man and the earth on which he is standing, instead of running through the circuit including the lamps. The effects are appalling—the whole of the muscles pass instantaneously into tonic contraction, so that the man remains clasping the wire, and cannot be removed from it whilst the current is passing. Sometimes a comrade also perishes owing to his trying to cut the wire to stop the current. The resistance which the body offers to the powerful current causes the development of great heat along its course; this is especially marked in the part of greatest resistance, which, as I have said before, is the skin. In many cases the whole hand has been burnt to a cinder, or deep holes have been burnt in the skin. Death is generally very rapid, but persons have lived twenty minutes after the accident, and few have recovered with permanent paralysis. One hundred and sixteen deaths are reported to have occurred in the United States from this cause in nine years, and many also in our own country. In America, as is known, this mode of death is employed for the execution of criminals, and the signs and lesions produced are reported to be identical with those produced accidentally. Deaths from this cause will, I am afraid, become more frequent unless special attention is directed to their prevention, since the insulating material tends to decay with age, and its insulating power becomes therefore diminished. It was also stated in evidence at an inquest a short time ago that rats frequently gnawed through the insulating material of wires. The workmen are ordered to wear indiarubber insulating gloves, which they often neglect to do; but this precaution would not always prevent accidents, since the current may enter at any part of the body which comes in contact with the wire, especially if the clothes are damp, so that their insulating power is diminished. These cases differ from deaths from lightning, in the fact that the lightning discharge enters and emerges from the body at several points, and hence the great burning and charring of skin and tissues are absent in death from this cause. The causes of death in these accidents are (1) asphyxia from tonic contraction of the respiratory muscles, (2) from disorganisation of the tissues by the passage of the current. On post-mortem examination the blood is found to be fluid everywhere, the protoplasm of the cells of the deeper layers of the skin is melted into a jelly-like material with numerous small spaces in it, which are probably bubbles of gas produced by electrolysis. By these appearances the lesions can be distinguished from ordinary burns, with which the superficial appearances of the affected skin are identical. The nervous tissues and muscles are found to be broken down into a granular material, and in many cases the large vessels have burst owing to development of gas in them from electrolysis of the blood. The recognition of this class of injuries is of great importance in forensic medicine, for, no doubt, murder might be committed by pushing anyone against an electric light conductor which was not properly insulated, and a body which had been artificially burnt might be placed near an electric conductor in order to suggest that the murdered person had been killed by the current. In these cases of accidental death from electricity, so far as I am aware no treatment has been tried hitherto, but the treatment suggested would be to employ artificial respiration to remove the condition of asphyxia, and to immerse the patient in a cold bath, or cover him with a wet sheet sprinkled with pounded ice, in order to remove the excess of heat produced in the body.

There is another class of case which is also coming into prominence in connection with electric lighting, in which people complain of the constant vibration produced by dynamos at the generating stations. This vibration is conducted along the wires and is more troublesome the nearer you are to the central station. Persons subjected to this tremor complain of debility, restlessness, tremulousness, and sleeplessness. It is possible also that the rapid alterations in electrical conditions of the surrounding bodies, which must accompany the alteration in the strength of the current in the wire, may influence the health of persons. The tremor would also injure any delicate apparatus in premises affected by it.

